# *Talaromyces marneffei* Detected in the Peripheral Blood Smear of an Individual Without Known Immunocompromise

**DOI:** 10.4269/ajtmh.21-1278

**Published:** 2022-04-11

**Authors:** Hirokazu Kuroda, Hiroaki Nishioka

**Affiliations:** Department of Infectious Diseases, Kobe City Medical Center General Hospital, Kobe, Japan

**Figure 1.**
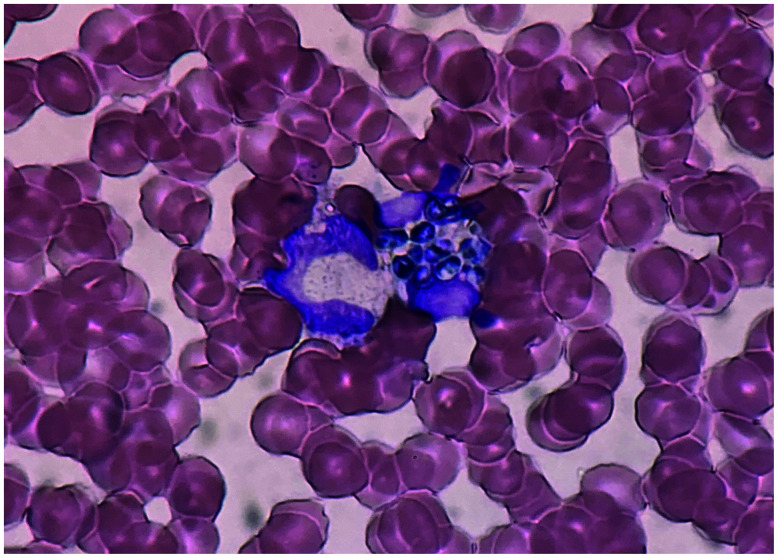
May-Grünwald-Giemsa–stained peripheral blood smear showing oval, yeast-like organisms within the cytoplasm of a neutrophil. This figure appears in color at www.ajtmh.org.

**Figure 2.**
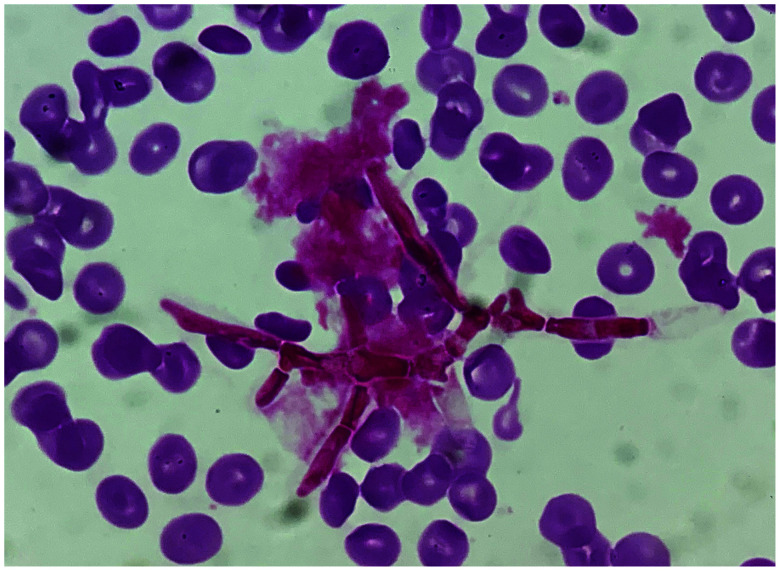
Gram-staining image of positive blood culture showing filamentous fungi. This figure appears in color at www.ajtmh.org.

A 24-year-old Vietnamese man suffering from fever along with bloody sputum for the past week and with a 4-month history of productive cough was admitted to our hospital. He has been studying in Japan for 2 years, but had visited Vietnam for 2 weeks, approximately 7 months ago. On admission, a computed tomography scan of the chest demonstrated bilateral, scattered lung infiltrates. Moreover, his May-Grünwald-Giemsa–stained peripheral blood smear exhibited oval, yeast-like organisms with a clearly defined central septum within the cytoplasm of a neutrophil (Figure 1). The sputum examinations on admission were negative for acid-fast bacilli smear and polymerase chain reaction assay of *Mycobacterium tuberculosis* and *Mycobacterium avium* complex. Therefore, we suspected disseminated *Talaromyces marneffei* infection and started him on liposomal amphotericin B therapy at 5 mg/kg daily. On the following day, we received reports of the blood cultures obtained on the day of admission, which confirmed the presence of filamentous fungi (Figure 2); 3 days later, these were identified as *T. marneffei*. Post-admission, his condition deteriorated rapidly, and he developed multiple organ failure, which was further complicated by hospital-acquired pneumonia and bacteremia caused by *Enterobacter cloacae* and *Klebsiella pneumoniae*. Despite aggressive treatment in the intensive care unit, he died on the eleventh day after hospitalization. Incidentally, there was no evidence of immunodeficiency diseases, including negative HIV serology, normal lymphocyte count, and normal immunoglobulin levels, in the investigations performed during the hospitalization period. Autoantibodies to interferon gamma were not involved in our investigation because they were commercially unavailable in Japan.

*Talaromyces marneffei*, formerly known as *Penicillium marneffei*, causes a fatal fungal infection, particularly in immunocompromised patients, such as those with HIV infection, and it is endemic to Southeast Asia and other tropical areas of Asia.^1^ However, it is extremely rare to find this fungal pathogen causing disseminated infection in immunocompetent patients.^2^ Anti-interferon-gamma autoantibody-associated immunodeficiency syndrome is a rare but one of the acquired immunodeficiency diseases associated with *T. marneffei* infection in Southeast Asia.^3^ Therefore, autoantibodies to interferon gamma as well as HIV serology should be investigated in the infected patients with no history of immunodeficiency diseases, if possible. In conclusion, as observed in this patient, a microscopic examination of peripheral blood smears, accompanied with a history of staying in endemic areas, may be useful for early presumptive diagnosis and subsequent treatment of disseminated *T. marneffei* infection, even in patients without preexisting immunocompromised conditions.
